# Risk factors, management, and future fertility of empty follicle syndrome: a retrospective study with real-world data

**DOI:** 10.3389/fendo.2024.1424837

**Published:** 2024-07-11

**Authors:** Zhuoye Luo, Suxin Xu, Guimin Hao

**Affiliations:** ^1^ Department of Reproductive Medicine, The Second Hospital of Hebei Medical University, Shijiazhuang, China; ^2^ Hebei Center for Quality Control and Management of Human Assisted Reproductive Technology, Shijiazhuang, China; ^3^ Hebei Key Laboratory of Infertility and Heredity, Shijiazhuang, China; ^4^ Hebei Clinical Research Center for Birth Defects, Shijiazhuang, China

**Keywords:** empty follicle syndrome, oocyte trigger, hCG exposure time, polycystic ovary syndrome, cumulative live birth rate

## Abstract

**Background:**

Empty follicle syndrome (EFS) is a challenging clinical problem. This study aims to identify the risk factors for EFS, to present pregnancy outcomes in both EFS cycle as well as subsequent cycles, and to summarize an effective rescue protocol to improve outcomes.

**Methods:**

A retrospective analysis between 2016 and 2020 was conducted at our center. Stricter criteria were applied to diagnose EFS. Logistic regression analysis was used to identify the risk factors for EFS. Further analyses were performed within the EFS cycle to present pregnancy outcomes and to find optimal rescue protocols. Long-term follow-up was conducted until live birth was achieved, covering at least two complete oocyte retrieval cycles.

**Results:**

Among 14,066 patients, 54 (0.38%) were identified as EFS. Patients with polycystic ovary syndrome (PCOS) had a significantly higher risk of developing EFS than non-PCOS ones (aOR = 2.67; 95% CI, 1.47 to 4.83). Within EFS patients, delaying the second oocyte retrieval by 3–6 h significantly improved the rates of obtaining oocyte (97.4% *versus* 58.3%, *P* = 0.002), getting embryo available for transfer (92.3% *versus* 33.3%, *P* < 0.001), and pregnancy (48.7% *versus* 8.3%, *P* = 0.017) compared to other delayed retrieval times. Overall, 31.5% (17/54) and 46.7% (7/15) EFS patients achieved live birth in the EFS cycle and the future cycle, respectively.

**Conclusions:**

PCOS is an independent risk factor for EFS, indicating that longer exposure time to human chorionic gonadotropin (hCG) may be necessary. Delaying the second oocyte retrieval by 3–6 h is an effective rescue protocol for EFS patients to achieve optimal outcomes. EFS in a single cycle does not necessarily indicate future fertility decline, but repeated EFS may result in poor outcomes.

## 1 Introduction

Empty follicle syndrome (EFS) was first described by Coulam et al. in 1986 (1). It is characterized by the failure to retrieve oocytes during repeated follicular aspiration and flushing despite appropriate follicular development and estradiol levels. The prevalence of EFS varies widely, as there is debate over whether low ovarian response patients should be included. Some studies define EFS as a no-oocyte obtained status that fits all patients (2, 3). However, other studies recommend excluding low responders from the definition of EFS (4, 5). They argue that low ovarian response is a sign of ovarian aging and a lower oocyte yield can be anticipated, thus justifying oocyte retrieval failure as a reasonable outcome rather than an indicator of EFS (6, 7). With stricter criteria, the incidence of EFS is estimated to be between 0.045% and 0.59% of cycles (4, 8, 9). Although rare, EFS can cause significant psychological distress for both healthcare providers and patients. Therefore, it is a challenging clinical problem that requires further investigation.

EFS is classified into genuine EFS (GEFS) and false EFS (FEFS) according to whether it has optimal beta human chorionic gonadotropin (β-hCG) levels after hCG injection. FEFS with negligible β-hCG levels indicates the possibility of injection mistake or pharmaceutical problem. However, no consensus has been reached on the etiologies of GEFS. The reasons are commonly speculated to be hCG factor (inadequate hCG exposure time, dosage, or activity) (10), inadequate ovarian response to hCG (11), and genetic or gene mutation factors (12, 13).

Due to the extremely low incidence of EFS, most original studies were reported as case reports. While the meta-analysis or reviews compensated for the inadequate sample size of each study, the significant heterogeneity of each study made it challenging to draw consistent and convincing conclusions about the risk factors, preventive measures, rescue protocols, and prognosis of EFS.

In this study, we applied rigorous EFS diagnostic criteria to analyze a large-sample dataset, aiming at present the risk factors for EFS and pregnancy outcomes in both EFS cycle as well as subsequent cycles. We also aim to summarize an effective rescue protocol to improve oocyte obtainment and pregnancy outcome.

## 2 Materials and methods

### 2.1 Study design and patients

This study was approved by the ethics committee of the Second Hospital of Hebei Medical University (No. 2024-R106). There is no requirement for informed consent.

A retrospective analysis was conducted on 21,567 cycles of oocyte retrieval performed in our center between January 2016 and December 2020. The study included patients with adequate follicular development in their gonadotropin-releasing hormone (GnRH) antagonist or GnRH agonist downregulation protocols triggered using hCG. Adequate follicular development was regarded as the presence of at least four follicles with a diameter of ≥14 mm, including at least two follicles with a diameter of ≥18 mm on the trigger day (4). EFS was defined as no oocyte obtained after repeated aspiration and flushing despite adequate follicular development. Repeated cycles, cycles with female abnormal chromosomes, and those who underwent preimplantation genetic testing (PGT) were excluded. Repeated cycles refer to multiple cycles with oocyte retrieval for one patient during the analysis. Finally, 14,066 patients were eligible, and among them, 54 cases of EFS were identified.

### 2.2 Procedures

Ovarian stimulation was routinely performed as we have mentioned previously (14). Oocyte retrieval was initiated 36–38 h after administering 6,500–10,000 IU of hCG (which included urinary hCG (u-hCG) and/or recombinant hCG (r-hCG); 250 µg of r-hCG was equivalent to 6,500 IU of u-hCG) to induce ovulation when at least two follicles with a diameter of ≥18 mm were present. Insemination was determined based on the infertility reason. Embryos got transferred or vitrified cryopreservation 3–5 days following oocyte retrieval. Embryo culture and luteal phase support were routinely conducted (15).

### 2.3 Rescue protocol

Oocyte retrieval was interrupted immediately if no oocyte was obtained after thorough aspiration and flushing during unilateral or six to eight follicles ≥14 mm in mean diameter puncture. Urinary β-hCG was then tested (8). If the urinary β-hCG was positive, and the average E_2_ levels per follicle ≥14 mm was less than 200 pg/mL as well as the number of such follicles was less than 15 on the trigger day, an additional 2,000–4,000 IU of hCG was administered, or there was no additional hCG injection otherwise. The second oocyte retrieval was delayed, varying from 1.6 to 7.1 h depending on the actual situation. If the urinary β-hCG was negative, blood β-hCG was tested to determine rescue hCG injection dosage and the second oocyte retrieval time. When no oocytes were retrieved during the EFS cycle, change in stimulation protocol, higher hCG dosage, and longer hCG exposure time were considered in the next cycle.

### 2.4 Pregnancy outcomes

Clinical pregnancy was confirmed by visualization of an intrauterine gestational sac with transvaginal ultrasound 4 to 5 weeks after embryo transfer. Miscarriage was defined as suffering pregnancy loss before 28 weeks of gestation after achieving clinical pregnancy. Live birth was defined as delivering at least one living child.

### 2.5 Statistical analysis

SPSS version 26.0 software package (SPSS Inc., Chicago, IL, USA) was used for statistical analysis. Continuous variables were expressed as mean ± standard deviation (mean ± SD) or median ± quartile range (median ± QR) according to distribution, with Student’s *t*-test or paired *t*-test for comparison in normal distribution and Mann–Whitney *U*-test or Wilcoxon paired test for comparison in non-normal distribution. Pearson’s chi-square analysis or Fisher’s exact test was applied in categorical variables. Logistic regression analysis was conducted to manifest the risk factors for EFS. Univariate logistic regression was conducted, and five variables with *P*-value less than 0.1 were included in the multivariate analysis. The sample size of EFS was 51, which met the minimum standard of 10 events per variable (EPV) to fit a model (16). When performing self-controlled comparison between EFS cycles and their normal cycles, paired *t*-test or Wilcoxon paired test was used to compare continuous variables and McNemar’s test was used to compare categorical variables. A two-sided *P*-value <0.05 means statistically significant.

## 3 Results

**Figure 1:**
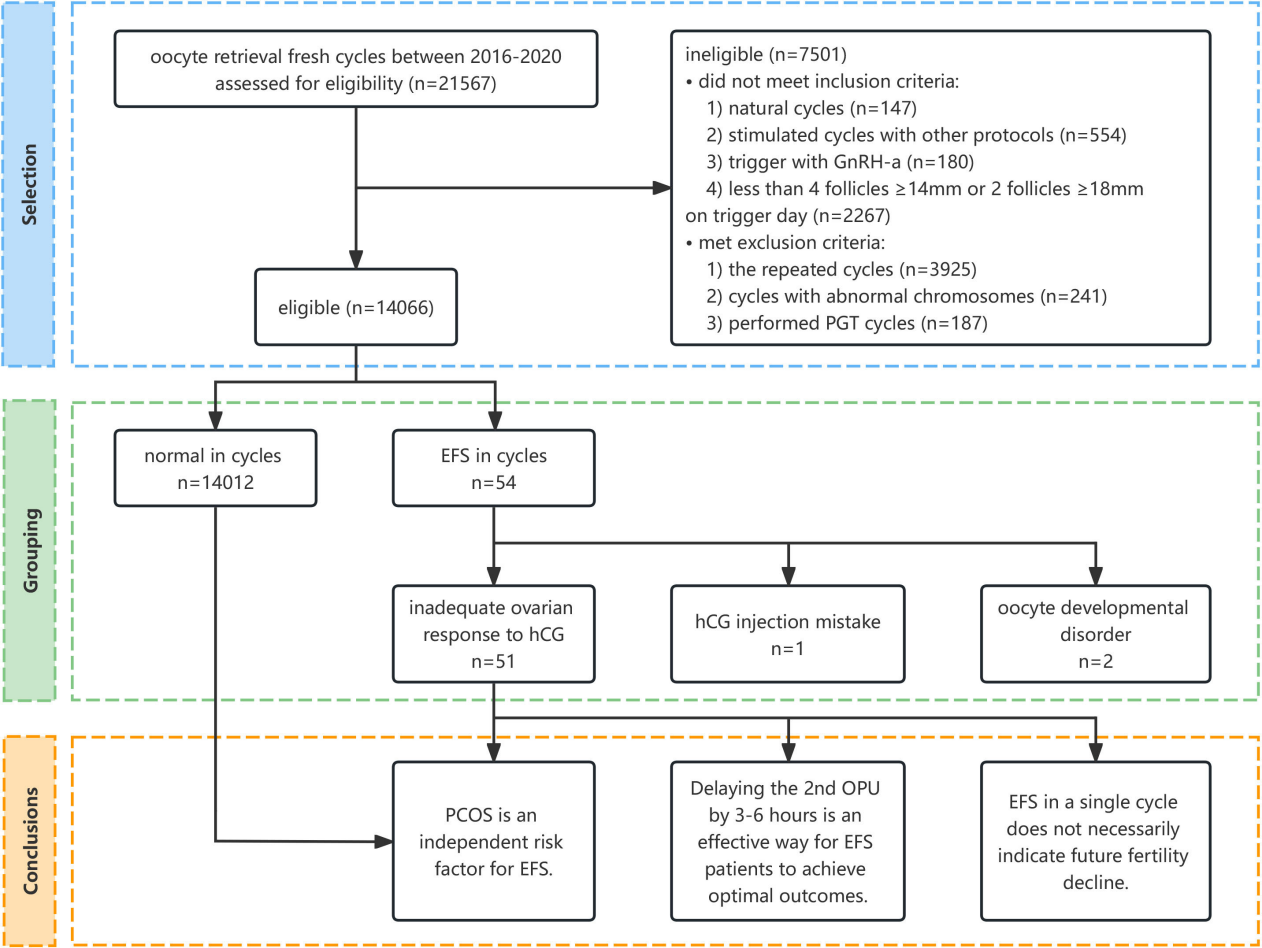
Flow diagram of the selection process and conclusion. GnRH-a, gonadotropin-releasing hormone agonist; PGT, preimplantation genetic testing; EFS, empty follicle syndrome; hCG, human chorionic gonadotropin; PCOS, polycystic ovary syndrome; OPU, oocyte pick-up.

A total of 14,066 patients were eligible, and 54 (0.38%) of them were identified as EFS (**Figure 1**).

### 3.1 Etiology of EFS

The causes of EFS were analyzed according to clinical manifestations and medical history. Specifically, one case was attributed to an hCG injection mistake, two cases were due to oocyte developmental disorder, and inadequate ovarian response to hCG was suspected in 51 other cases (**Figure 1**) (**Supplementary Table S1**).

Urinary β-hCG was found to be negative in only one patient. On the day of oocyte retrieval, her blood levels of β-hCG, luteinizing hormone (LH), and progesterone (P) were 1.4 IU/L, 4.58 IU/L, and 1.38 ng/mL, respectively. The patient self-reported a shallow hCG injection with the skin surface moist. We categorized this as an hCG injection error, and a second oocyte retrieval was performed 36 h later following the administration of 10,000 IU of hCG for rescue. The patient successfully obtained oocytes and achieved a live birth.

Two patients had suffered EFS during a previous cycle. All oocytes obtained were in metaphase I (MI) stage with abnormal zona pellucida. They were both primary infertility cases and denied a family history of infertility. We classified the condition as oocyte developmental disorder and suspected genetic abnormalities, but the patients declined further genetic testing.

We could not find an obvious reason for EFS in the other 51 cases, so they were classified into inadequate ovarian response to hCG tentatively.

### 3.2 Risk factors for EFS

The EFS group had a higher body mass index (BMI) (25.3 ± 4.4 *versus* 23.6 ± 3.6, *P* = 0.001), a higher proportion of PCOS (45.1% *versus* 18.6%, *P* < 0.001), a higher proportion of downregulated protocols (86.3% *versus* 77.7%, *P* = 0.004), and a lower E_2_ per ≥14-mm follicle (265.5 ± 99.3 *versus* 333.4 ± 118.9, *P* < 0.001) than the group with normal situation for oocyte retrieval (non-EFS group). However, there were no significant differences in other factors between the two groups. After adjusting for confounding factors, PCOS was found to be a significant risk factor for EFS (aOR = 2.67; 95% CI, 1.47 to 4.83) (**Table 1**).

**Table 1:** Risk factors for EFS.

	Non-EFS (*n* = 14,012)	EFS (*n* = 51)^a^	Crude OR (95% CI)	*P*	Adjusted OR (95% CI)	*P*
Age (years)	30.5 ± 4.2	29.9 ± 3.1	0.97 (0.90, 1.00)	0.315	–	–
BMI (kg/m^2^)	23.6 ± 3.6	25.3 ± 4.4	1.13 (1.05, 1.21)	0.001	1.07 (0.99, 1.16)	0.064
Basal FSH (IU/L)	7.1 ± 2.2	6.7 ± 1.4	0.91 (0.79, 1.05)	0.192	–	–
Basal LH (IU/L)	6.9 ± 3.3	7.4 ± 4.1	1.04 (0.96, 1.12)	0.315	–	–
Infertility types				0.790	–	–
Primary infertility	7,707 (55.0%)	29 (56.9%)	Reference			
Secondary infertility	6,305 (45.0%)	22 (43.1%)	0.93 (0.53, 1.62)			
Infertility factors				<0.001		0.001
Non-PCOS	11,400 (81.4%)	28 (54.9%)	reference		reference	
PCOS	2,612 (18.6%)	23 (45.1%)	3.59 (2.06, 6.23)		2.67 (1.47, 4.83)	
Protocols				0.004		0.164
GnRH-ant	3,122 (22.3%)	7 (13.7%)	reference		reference	
GnRH-a long	5,023 (35.8%)	30 (58.8%)	2.66 (1.17, 6.07)		2.07 (0.89, 4.79)	
GnRH-a short	5,867 (41.9%)	14 (27.5%)	1.06 (0.43, 2.64)		1.31 (0.52, 3.26)	
Gn dosage per day (IU)	232.4 ± 74.9	230.1 ± 60.7	1.00 (0.99, 1.01)	0.784	–	–
E_2_ per ≥14-mm follicle (pg/mL)	333.4 ± 118.9	265.5 ± 99.3		<0.001		0.065
<200	1,789 (13.3%)	14 (27.5%)	2.47 (1.34, 4.59)		1.84 (0.96, 3.50)	
≥200	11,697 (86.7%)	37 (72.5%)	Reference		Reference	
hCG types				0.376	–	–
Urinary hCG	4,411 (31.5%)	19 (37.3%)	Reference			
Recombined hCG	9,601 (68.5%)	32 (62.7%)	0.77 (0.44, 1.37)			
hCG dosage (IU)/1,000	7.7 ± 1.1	8.0 ± 1.2	1.26 (0.99, 1.61)	0.063	1.26 (0.98, 1.60)	0.067
hCG exposure time (h)	36.6 ± 0.7	36.5 ± 0.5	0.76 (0.52, 1.11)	0.152	–	–

Data are presented as either means ± SD or number (%).

EFS, empty follicle syndrome; BMI, body mass index; FSH, follicle-stimulating hormone; PCOS, polycystic ovary syndrome; GnRH-a, gonadotropin-releasing hormone agonist; Gn, gonadotropin; hCG, human chorionic gonadotropin.

a Only EFS cases due to inadequate ovarian response to hCG were included.

### 3.3 Optimal delayed time for second oocyte retrieval

We classified the delayed time by each hour and analyzed the clinical outcomes of each group. Subsequently, we merged the groups into two categories, namely, the 3–6h group and the non-3–6h group. Although the demographic data and the number of ≥14 mm follicles on the trigger day were similar in both groups, patients in the 3–6h group showed a significantly higher rate of obtaining oocyte (97.4% *versus* 58.3%, *P* = 0.002), obtaining embryo available for transfer (92.3% *versus* 33.3%, *P* < 0.001), and achieving pregnancy (48.7% *versus* 8.3%, *P* = 0.017) compared with those in the non-3–6h group (**Table 2**).

**Table 2:** Clinical outcomes during the empty follicle syndrome cycle of patients with different delayed time for the second oocyte retrieval.

Variables	Delayed time (h)					Group of delayed time (h)		
	<3	[3, 4]	(4, 5]	(5, 6]	>6	<3 or >6	[3, 6]	*P*
N*	9	11	19	9	3	12	39	–
Age (years)	30.6 ± 2.1	30.3 ± 3.7	30.0 ± 3.2	29.6 ± 2.6	27.0 ± 4.0	29.7 ± 3.0	30.0 ± 3.2	0.747
BMI (kg/m^2^)	25.3 ± 4.3	24.4 ± 4.0	26 ± 4.8	24.9 ± 4.3	24.7 ± 5.9	25.2 ± 4.4	25.3 ± 4.4	0.924
Basal FSH (IU/L)	6.9 ± 1.5	6.4 ± 1.2	6.6 ± 1.5	7.2 ± 1.4	7.1 ± 1.3	7.0 ± 1.4	6.7 ± 1.4	0.532
No. of ≥14-mm follicles on trigger day	10.9 ± 2.2	14.7 ± 4.4	12.8 ± 5.7	12.4 ± 4.4	14.3 ± 4.9	11.8 ± 3.2	13.3 ± 5.0	0.334
Patients with oocyte obtained (%)	5 (55.6)	11 (100.0)	18 (94.7)	9 (100.0)	2 (66.7)	7 (58.3)	38 (97.4)	0.002
Patients with embryo for transfer (%)	3 (33.3)	11 (100.0)	18 (94.7)	7 (77.8)	1 (33.3)	4 (33.3)	36 (92.3)	<0.001
No. of pregnancies (%)	0 (0.0)	5 (45.5)	9 (47.4)	5 (55.6)	1 (33.3)	1 (8.3)	19 (48.7)	0.017

Data are presented as either means ± SD or number (%). Continuous variables were compared using Student’s t test. Categorical variables were compared using Exact Fisher test.

*Only EFS cases due to inadequate ovarian response to hCG were included.

### 3.4 Self-controlled comparison between EFS cycles and their normal cycles

A total of 16 EFS patients who had previously normal cycles were analyzed. The protocols, E_2_ per ≥14 mm follicle, hCG dosage, and exposure were all similar between the previous normal cycle and EFS cycle, but the EFS cycle showed significantly lower numbers of oocyte obtained (4.3 ± 3.8 *versus* 9.4 ± 5.3, *P* = 0.003), two polar nucleus (PN) embryos (3.2 ± 3.0 *versus* 5.5 ± 3.2, *P* = 0.021), and embryos available for transfer (1.3 ± 1.2 *versus* 1.9 ± 1.1, *P* = 0.023) (**Table 3**).

**Table 3:** Self-controlled comparison between EFS cycles and their future normal cycles.

Variables	Previous normal cycle (*n* = 16)	EFS cycle (*n* = 16)	*P*	EFS cycle (*n* = 15)	Subsequent normal cycle (*n* = 15)	*P*
Age (years)	29.5 ± 3.1	30.4 ± 2.4	0.184	30.8 ± 3.0	31.5 ± 3.2	0.012
Downregulated protocol	13 (81.3%)	11 (68.8%)	0.727	13 (86.7%)	2 (13.3%)	0.001
hCG dosage (IU)	8,156 ± 1,044	8,438 ± 1,276	0.402	8,167 ± 1,249	8,800 ± 1,811	0.270
hCG exposure time (IU)	36.4 ± 0.3	36.3 ± 0.4	0.253	36.5 ± 0.5	38.0 ± 1.2	<0.001
E_2_ per ≥14-mm follicle (pg/mL)	328.1 ± 94.4	339.6 ± 101.6	0.778	264.1 ± 101.7	336.1 ± 106.0	0.062
No. of oocytes	9.4 ± 5.3	4.3 ± 3.8	0.003	3.7 ± 2.7	10.5 ± 4.6	0.001
No. of 2PN embryos	5.5 ± 3.2	3.2 ± 3.0	0.021	2.5 ± 2.2	6.0 ± 3.7	0.006
No. of embryos for transfer	1.9 ± 1.1	1.3 ± 1.2	0.023	1.1 ± 0.9	2.9 ± 2.5	0.006
Clinical pregnancy rate	3 (18.8%)	3 (18.8%)	1.000	1 (6.7%)	8 (53.3%)	0.039
Live birth rate	3 (18.8%)	2 (12.5%)	1.000	0 (0%)	7 (46.7%)	0.016

Data are presented as either means ± SD or number (%). Continuous variables were compared using paired t-test or Wilcoxon paired test. Categorical variables were compared using McNemar’s test.

EFS, empty follicle syndrome; hCG, human chorionic gonadotropin; PN, polar nucleus.

**Figure 2:**
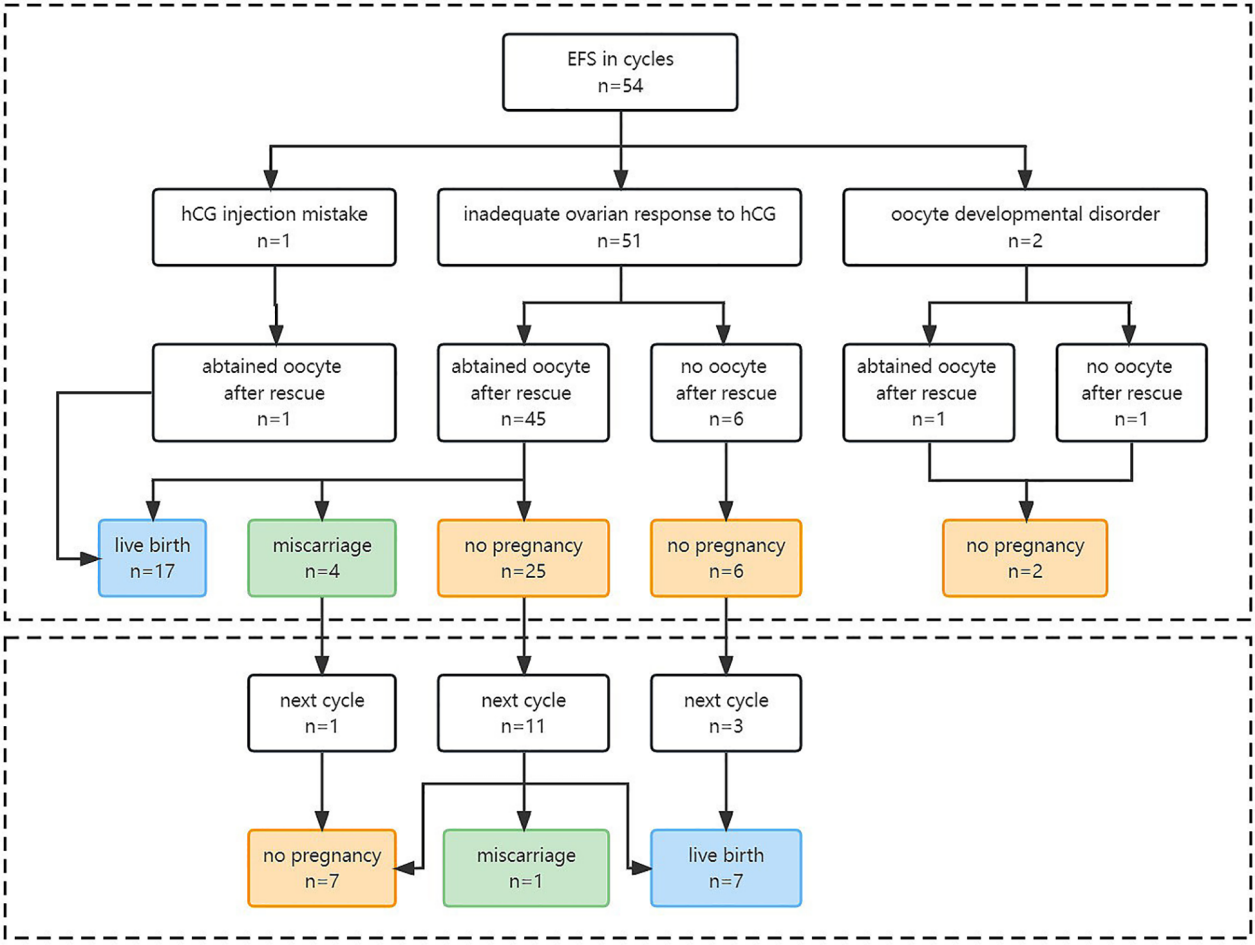
Pregnancy outcomes of EFS patients in EFS cycle and the future cycle. EFS, empty follicle syndrome; hCG, human chorionic gonadotropin.

Moreover, 15 EFS patients who failed to achieve live birth in their EFS cycle underwent a subsequent normal cycle, which showed significantly higher numbers of oocytes obtained (10.5 ± 4.6 *versus* 3.7 ± 2.7, *P* = 0.001), two PN embryos (6.0 ± 3.7 *versus* 2.5 ± 2.2, *P* = 0.006), and embryos available for transfer (2.9 ± 2.5 *versus* 1.1 ± 0.9, *P* = 0.006). No patient repeated EFS after performing less downregulated protocol and longer hCG exposure time. The live birth rate in the subsequent normal cycle was 46.7% (7/15) (**Table 3**) (**Figure 2**).

## 4 Discussion

Our study indicated that PCOS patients are more prone to EFS and may require a longer hCG exposure for ovulation. Delaying the second oocyte retrieval by 3–6 h may be an effective way to achieve optimal pregnancy outcomes in EFS cases. EFS that occurred once did not suggest a fertility decline in future cycles.

### 4.1 Etiology of EFS

Studies have shown that mature metaphase II (MII) oocytes can be obtained 28–38 h after the onset of LH peak, and hCG exposure time less than 36 h significantly decreases the oocyte number and maturity (17, 18). The optimal interval time remains unclear, but 36–38 h is widely accepted (19, 20). Our center administered a minimum hCG dosage of 6,500 IU to induce ovulation, surpassing the recommended minimum dosage of 5,000 IU in previous studies (21). Thus, the hCG exposure time and dosage both met the routine clinical criteria for all patients.

An accidental hCG injection mistake, as seen in our study, can halt ovarian stimulation without triggering ovulation, which was similar to “coasting”. Previous studies showed that 1 to 2 days of “coasting” is harmless (22, 23). Stevenson reported live births in six out of 14 similar cases after rescue hCG injection, suggesting that oocyte and embryo quality may not be compromised (24). However, another study presented an increased rate of embryonic triploidy and compromised outcomes after rescue (4). Additionally, we should pay attention to spontaneous LH surge in GnRH-antagonist protocol. Blood LH and P levels should be tested after EFS to determine the appropriate time for the second oocyte retrieval.

Repeated EFS may be associated with premature oocyte atresia or oocyte maturation disorders, with the oocytes obtained presented as germinal vesicle (GV) or MI stage (25), immature oocytes without zona pellucida (26), or with an identifiable zona but devoid of oocytes (27). Our study identified two patients with repeated EFS, whose oocytes were all in the MI stage with abnormal zona pellucida, consistent with previous findings. GnRH-a triggering can stimulate FSH surge simultaneously, and dual trigger combined with hCG may be an alternative to improve oocyte maturation (28, 29). However, these two patients failed to obtain mature oocyte by dual triggering in previous EFS cycles. These suggested that they possibly had oocyte developmental disorders related to genetic factors, such as luteinizing hormone/choriogonadotropin receptor (LHCGR) (12) or zona pellucida (ZP) subtype (13).

However, most EFS cases in our study lacked an obvious cause. We speculated that individual hCG thresholds vary, and routine hCG dosage/exposure time may be insufficient for patients with higher thresholds, resulting in EFS. Additional HCG injections or extended exposure to HCG may improve oocyte retrieval, indicating inadequate or delayed response to HCG in these patients. Blazquez et al. also mentioned similar cases and hypothesized that EFS in these patients might be attributed to temporary signal conduction delay rather than an ovarian pathological problem (8). Experimental evidence is needed to prove this speculation further.

### 4.2 Risk factors for EFS

PCOS patients were found to be more susceptible to EFS, possibly due to a persistently higher LH level and the inadequate or delayed expression of LH receptors. Thus, they may require more dosage of or exposure time to hCG to be triggered. Previous studies have supported this conclusion (11, 30). Daichi et al. speculated that the significantly fewer oocytes collected from the group of patients with higher LH were due to their insufficient FSH receptor (31).

Gambini et al. found that a higher BMI was associated with an increasing risk of oocyte immaturity after GnRH-a triggering (32). Pharmacokinetics changes associated with high BMI may partly explain the difference (33). In our study, BMI was significantly higher in the EFS group; however, the difference failed to reach statistical significance after adjustment. Singh et al. suggested a higher EFS occurrence in GnRH-antagonist protocols (34), but other studies have shown no impact of stimulation protocol on EFS prevalence (5, 35). Our findings align with the latter despite a higher proportion of EFS cases in the downregulated protocol. Differences in race and EFS criteria may account for this discrepancy, necessitating further investigation.

### 4.3 Rescue protocol

E_2_ per mature follicle typically ranges from 200 to 300 pg/mL before ovulation (36), and lower levels indicate oocyte immaturity and poor prognosis. Thus, in EFS cases with E_2_ less than 200 pg/mL per follicle, an additional 2,000–4,000 IU of hCG was administered. To reduce the risk of ovarian hyperstimulation, patients with over 15 follicles ≥14 mm were not given additional hCG (37).

It was reported that delaying the second retrieval by over 6 h can rescue 70% of EFS cases (38), but we found that delaying by 3–6 h help in achieving optimal outcomes. Too short or long of a delay may result in retrieval failure. Discrepancies in results may be due to differences in trigger standards and race.

### 4.4 Pregnancy outcomes in EFS cycles and the future cycles

Our study found that 31.5% (17/54) of EFS patients achieved live birth in the same cycle. For those who had a failed pregnancy in the EFS cycle, altering stimulation protocols, increasing hCG dosage or exposure time, or using a dual trigger of hCG combined with GnRH-a (39, 40) in the next cycle resulted in a live birth rate of 46.7% (7/15). This rate was comparable to the overall cumulative live birth rate of one retrieval cycle for all patients (50%) in our center. Thus, we speculated that the compromised outcome in the EFS cycle maybe due to the insufficient oocyte number obtained rather than fertility decline. Our findings are consistent with Revelli’s opinion (35) but in conflict with Lorusso’s (41), which found in three patients that EFS could predict less optimistic outcomes of the subsequent cycle. Larger studies are needed to resolve this discrepancy.

Obtaining a favorable pregnancy outcome with repeated EFS is challenging (42). *In vitro* maturation offers promise for patients with oocyte maturation problems (43, 44), while oocyte donation is a last-resort option.

### 4.5 Strength and limitations

This single-center study had a large sample size and low heterogeneity. It is the first study to analyze the risk factors for EFS with hCG trigger using multivariate logistic regression, and patients with PCOS were found to be more susceptible to EFS. We just found that delaying the second retrieval for 3–6 h may be an effective way for EFS to achieve optimal outcomes. Additionally, our long-term follow-up, including at least two complete oocyte retrieval cycles until live birth, is a novel contribution not mentioned in previous studies. However, the retrospective nature of our study may limit its statistical power due to potential biases and incomplete data. Further studies are required to confirm our findings and provide stronger evidence.

## 5 Conclusion

PCOS is an independent risk factor for EFS, possibly requiring a longer hCG exposure time. Delayed second oocyte retrieval by 3–6 h is an effective way for EFS to achieve optimal outcomes. The occurrence of EFS in a single cycle does not necessarily indicate future fertility decline, but repeated instances of EFS are associated with poor outcomes.

## Data availability statement

The raw data supporting the conclusions of this article will be made available by the authors, without undue reservation.

## Ethics statement

The studies involving humans were approved by the ethics committee of the Second Hospital of Hebei Medical University (No. 2024-R106). The studies were conducted in accordance with the local legislation and institutional requirements. Written informed consent for participation was not required from the participants or the participants’ legal guardians/next of kin because the study was retrospective and patients’ information was anonymous, which is in accordance with the national legislation and the institutional requirements.

## Author contributions

ZL: Funding acquisition, Software, Writing – original draft. SX: Conceptualization, Data curation, Methodology, Supervision, Writing – review & editing. GH: Conceptualization, Funding acquisition, Investigation, Methodology, Supervision, Validation, Writing – review & editing.
