# Supplementary material for: Risk factors, management, and future fertility of empty follicle syndrome: a retrospective study with real-world data
Source: Front Endocrinol (Lausanne). 2024 Jul 11;15:1424837. doi: 10.3389/fendo.2024.1424837 (PMC11269657; doi:10.3389/fendo.2024.1424837)
Supplement: Supplementary file 1 [file Table_1.docx]

Supplementary Material

# Supplementary Table S1

Table S1 Demographic, clinical and laboratory characteristics, and clinical outcomes of patients suffered EFS.

| No. | age | Infertility factor | Cycle No. | protocol | Gn total dosage (IU) | E2 per ≥14mm follicle (pg/ml) | hCG type and dosage (IU) | hCG exposure time (h) | Urinary or blood β-hCG (IU/L) | Rescue u-hCG dosage (IU) | Delayed time (h) | No. of oocyte / follicles# | No. of embryo for ET | Pregnancy outcomes | EFS reason |
| --- | --- | --- | --- | --- | --- | --- | --- | --- | --- | --- | --- | --- | --- | --- | --- |
| 1 | 32 | Oviduct | 2 | GnRH-a long | 3075 | 312 | r-6500 | 36.5 | Positive | - | 4.0 | 6/14 | 2 | Live birth | inadequate ovarian response to hCG |
| 2 | 29 | Oviduct | 2 | GnRH-ant | 2100 | 206 | u-8000 | 36.1 | Positive | - | 2.0 | 5/9 | 2 | No pregnancy | inadequate ovarian response to hCG |
| 3 | 28 | Oviduct | 1 | GnRH-a short | 3000 | ＞361 | u-8000 | 36.7 | Positive | - | 2, 5.2 | 0/14 | - | Live birth* | inadequate ovarian response to hCG |
| 4 | 29 | PCOS | 1 | GnRH-a long | 2250 | 195 | r-6500+ u-2000 | 36.2 | Positive | - | 4.3 | 4/9 | 2 | No pregnancy | inadequate ovarian response to hCG |
| 5 | 33 | Oviduct | 1 | GnRH-a long | 1425 | 125 | r-6500+ u-2000 | 37.3 | Positive | 2000 | 2.3 | 5/14 | 2 | Live birth* | inadequate ovarian response to hCG |
| 6 | 24 | male | 1 | GnRH-a short | 2250 | ＞281 | r-6500 | 36.3 | Positive | - | 4.0 | 9/18 | 3 | Live birth | inadequate ovarian response to hCG |
| 7 | 31 | Oviduct | 1 | GnRH-a short | 2700 | 332 | u-10000 | 37 | Positive | - | 3.2 | 8/8 | 3 | No pregnancy | inadequate ovarian response to hCG |
| 8 | 28 | PCOS | 1 | GnRH-a long | 1350 | ＞337 | u-8000 | 36.5 | Positive | - | 4.0 | 10/15 | 6 | No pregnancy | inadequate ovarian response to hCG |
| 9 | 27 | Oviduct +PCOS | 1 | GnRH-a long | 1012 | ＞281 | r-6500 | 37.4 | Positive | - | 3.0 | 18/20 | 4 | Live birth | inadequate ovarian response to hCG |
| 10 | 30 | PCOS | 1 | GnRH-a long | 2000 | ＞253 | r-6500 | 36.7 | Positive | - | 3.2 | 6/20 | 5 | Live birth | inadequate ovarian response to hCG |
| 11 | 31 | Oviduct +PCOS | 1 | GnRH-a long | 4350 | 171 | r-6500 | 35.9 | Positive | 4000 | 6.7 | 0/11 | - | Live birth * | inadequate ovarian response to hCG |
| 12 | 29 | Oviduct | 2 | GnRH-a long | 3900 | ＞562 | r-6500+ u-2000 | 36.2 | Positive | - | 2.0 | 2/9 | 0 | No pregnancy | oocyte developmental disorder |
| 13 | 32 | PCOS | 1 | GnRH-ant | 2625 | ＞252 | r-6500 | 36.3 | 1.4 | 10000 | 36 | 8/18 | 5 | Live birth | hCG injection mistake |
| 14 | 31 | Unexplained | 2 | GnRH-ant | 2925 | 253 | u-10000 | 36.3 | Positive | - | 2, 5.3 | 0/9 | - | No pregnancy | inadequate ovarian response to hCG |
| 15 | 28 | PCOS | 2 | GnRH-a long | 1500 | 228 | u-9000 | 36.5 | Positive | - | 2, 22 | 0/11 | - | No pregnancy | inadequate ovarian response to hCG |
| 16 | 30 | Oviduct | 2 | GnRH-a short | 3112.5 | 263 | u-10000 | 35.6 | Positive | - | 4.5 | 8/8 | 2 | No pregnancy* | inadequate ovarian response to hCG |
| 17 | 38 | Oviduct | 1 | GnRH-a short | 1650 | >329 | r-6500+ u-2000 | 36.5 | Positive | - | 3.5 | 14/15 | 2 | No pregnancy | inadequate ovarian response to hCG |
| 18 | 31 | male | 2 | GnRH-a long | 3487.5 | >448 | u-8000 | 36.2 | Positive | - | 2.5 | 0/11 | - | No pregnancy | inadequate ovarian response to hCG |
| 19 | 29 | Oviduct +male | 1 | GnRH-a long | 5325 | 147 | u-8000 | 37.2 | Positive | 2000 | 2, 5.5 | 1/8 | 0 | No pregnancy | inadequate ovarian response to hCG |
| 20 | 31 | PCOS | 1 | GnRH-a short | 2062.5 | >197 | r-6500 | 36.2 | Positive | - | 5.0 | 11/25 | 3 | live birth | inadequate ovarian response to hCG |
| 21 | 29 | PCOS | 1 | GnRH-a long | 1125 | >197 | r-6500 | 36.3 | Positive | - | 4.5 | 23/25 | 7 | live birth | inadequate ovarian response to hCG |
| 22 | 29 | male | 1 | GnRH-a long | 2250 | >246 | r-6500 | 35.7 | Positive | - | 5.5 | 19/20 | 6 | live birth | inadequate ovarian response to hCG |
| 23 | 28 | PCOS | 1 | GnRH-a long | 2175 | 229 | r-6500 | 36.7 | Positive | - | 4.7 | 10/13 | 2 | No pregnancy | inadequate ovarian response to hCG |
| 24 | 26 | PCOS | 1 | GnRH-a long | 4350 | >274 | r-6500 | 37.5 | Positive | - | 4.5 | 15/18 | 1 | No pregnancy | inadequate ovarian response to hCG |
| 25 | 28 | Oviduct | 1 | GnRH-a short | 3125 | 184 | r-6500 | 36.7 | Positive | 2000 | 6.0 | 11/11 | 2 | live birth | inadequate ovarian response to hCG |
| 26 | 29 | Oviduct | 1 | GnRH-a short | 2100 | 170 | r-6500 | 36.3 | Positive | - | 3.0 | 17/20 | 4 | No pregnancy | inadequate ovarian response to hCG |
| 27 | 28 | Oviduct | 3 | GnRH-ant | 3200 | 250 | u-8000 | 36.7 | Positive | - | 3.0 | 0/10 | - | No pregnancy | oocyte developmental disorder |
| 28 | 29 | PCOS | 1 | GnRH-a long | 1650 | >379 | u-8000 | 37 | Positive | - | 4.2 | 9/13 | 3 | live birth | inadequate ovarian response to hCG |
| 29 | 35 | Oviduct | 2 | GnRH-a long | 3150 | 268 | u-8000 | 36.5 | Positive | - | 4.2 | 8/18 | 1 | live birth* | inadequate ovarian response to hCG |
| 30 | 30 | Oviduct | 1 | GnRH-a long | 4275 | 114 | r-6500+ u-2000 | 36.3 | Positive | 2000 | 4.5 | 9/12 | 2 | live birth | inadequate ovarian response to hCG |
| 31 | 33 | PCOS | 1 | GnRH-a long | 4800 | 187 | r-6500 +u-2000 | 37 | Positive | - | 2.1 | 4/12 | 1 | No pregnancy* | inadequate ovarian response to hCG |
| 32 | 28 | PCOS | 1 | GnRH-a long | 2325 | 248 | r-6500 +u-2000 | 37 | Positive | - | 4.4 | 6/10 | 2 | miscarriage- No pregnancy* | inadequate ovarian response to hCG |
| 33 | 24 | PCOS | 1 | GnRH-a long | 3300 | 146 | r-6500 +u-2000 | 35.8 | Positive | 2000 | 6.0 | 8/13 | 5 | live birth | inadequate ovarian response to hCG |
| 34 | 31 | PCOS | 1 | GnRH-a long | 1350 | 231 | u-9000 | 36.7 | Positive | - | 4.0 | 3/11 | 2 | live birth | inadequate ovarian response to hCG |
| 35 | 26 | PCOS | 1 | GnRH-a long | 2925 | 223 | r-6500 | 36.6 | Positive | - | 4.8 | 4/13 | 2 | live birth* | inadequate ovarian response to hCG |
| 36 | 31 | PCOS | 1 | GnRH-a long | 5250 | 202 | r-6500 +u-4000 | 36.2 | Positive | - | 5.2 | 17/18 | 3 | live birth | inadequate ovarian response to hCG |
| 37 | 34 | Endometriosis | 1 | GnRH-a long | 5850 | 113 | u-10000 | 36.9 | Positive | 2000 | 4.9 | 6/8 | 2 | No pregnancy* | inadequate ovarian response to hCG |
| 38 | 37 | Oviduct | 1 | GnRH-ant | 4875 | 212 | u-8000 | 36.5 | Positive | - | 4.3 | 3/8 | 2 | No pregnancy | inadequate ovarian response to hCG |
| 39 | 27 | PCOS | 1 | GnRH-a short | 1350 | 227 | r-6500 | 36.7 | Positive | - | 6.2 | 9/20 | 3 | live birth | inadequate ovarian response to hCG |
| 40 | 35 | PCOS | 1 | GnRH-a long | 3000 | 216 | r-6500 +u-2000 | 35.8 | Positive | - | 4.3 | 7/14 | 3 | live birth | inadequate ovarian response to hCG |
| 41 | 31 | Endometriosis | 1 | GnRH-a long | 2325 | 278 | r-6500 | 35.8 | Positive | - | 5.2 | 2/9 | 1 | live birth* | inadequate ovarian response to hCG |
| 42 | 23 | Oviduct | 1 | GnRH-a long | 2250 | 194 | r-6500 +u-2000 | 36.7 | Positive | - | 7.1 | 5/12 | 0 | No pregnancy | inadequate ovarian response to hCG |
| 43 | 33 | Oviduct | 1 | GnRH-ant | 1800 | 226 | r-6500 +u-2000 | 36.4 | Positive | - | 1.6 | 2/10 | 0 | miscarriage* | inadequate ovarian response to hCG |
| 44 | 30 | Oviduct | 1 | GnRH-a short | 2625 | 457 | u-8000 | 36 | Positive | - | 4.6 | 5/7 | 2 | miscarriage | inadequate ovarian response to hCG |
| 45 | 29 | Oviduct | 1 | GnRH-a long | 2850 | 144 | r-6500 +u-2000 | 36.8 | Positive | 2000 | 5.6 | 12/13 | 2 | miscarriage | inadequate ovarian response to hCG |
| 46 | 29 | PCOS | 2 | GnRH-a short | 1200 | 300 | u-9000 | 36.8 | Positive | - | 4.2 | 8/16 | 2 | No pregnancy | inadequate ovarian response to hCG |
| 47 | 29 | PCOS | 2 | GnRH-a short | 1575 | 480 | u-8000 | 35.8 | Positive | - | 3.0 | 2/10 | 1 | No pregnancy | inadequate ovarian response to hCG |
| 48 | 33 | PCOS | 2 | GnRH-ant | 4650 | 540 | r-6500 | 36.7 | Positive | - | 5.7 | 2/8 | 0 | No pregnancy | inadequate ovarian response to hCG |
| 49 | 30 | Unexplained | 3 | GnRH-a long | 2725 | 437 | r-6500 | 36.9 | Positive | - | 5.3 | 3/7 | 2 | No pregnancy* | inadequate ovarian response to hCG |
| 50 | 29 | Oviduct | 2 | GnRH-a long | 3375 | 327 | r-6500 +u-4000 | 35.8 | Positive | - | 4.5 | 13/14 | 3 | live birth | inadequate ovarian response to hCG |
| 51 | 31 | PCOS | 3 | GnRH-a short | 2750 | 372 | u-8000 | 36.4 | Positive | - | 5.4 | 5/13 | 0 | No pregnancy* | inadequate ovarian response to hCG |
| 52 | 31 | Oviduct | 2 | GnRH-ant | 2100 | 311 | r-6500 +u-2000 | 36.3 | Positive | - | 4.6 | 6/7 | 4 | miscarriage | inadequate ovarian response to hCG |
| 53 | 25 | Oviduct | 3 | GnRH-a short | 5587.5 | 243 | u-10000 | 36.4 | Positive | - | 4.7 | 0/5 | - | live birth* | inadequate ovarian response to hCG |
| 54 | 34 | Oviduct+ male | 2 | GnRH-ant | 2400 | 446 | r-6500 +u-2000 | 35.9 | Positive | - | 4.0 | 3/11 | 1 | No pregnancy* | inadequate ovarian response to hCG |
| EFS, empty follicle syndrome; PCOS, polycystic ovary syndrome; Gn, gonadotropin; hCG, human chorionic gonadotropin; GnRH-a, gonadotropin-releasing hormone agonist; GnRH-ant, gonadotropin-releasing hormone antagonist; r-hCG, recombinant hCG; u- hCG, urinary hCG.  * Pregnancy outcomes in the future subsequent cycle.  # “No. of oocyte / follicles” means “No. of oocyte obtained / No. of follicle ≥14mm on trigger day”. | | | | | | | | | | | | | | | |
